# The Effect of Simvastatin on Gut Microbiota and Lipid Metabolism in Hyperlipidemic Rats Induced by a High-Fat Diet

**DOI:** 10.3389/fphar.2020.00522

**Published:** 2020-04-29

**Authors:** Qing Zhang, Xiaoyun Fan, Rui Ye, Yuzhong Hu, Tingting Zheng, Rui Shi, Wenjian Cheng, Xucong Lv, Lijiao Chen, Peng Liang

**Affiliations:** ^1^College of Food Science, Fujian Agriculture and Forestry University, Fuzhou, China; ^2^Institute of Food Science and Technology, College of Biological Science and Technology, Fuzhou University, Fuzhou, China

**Keywords:** simvastatin, hyperlipidemia, lipid metabolism, metabolomics, gut microbiota

## Abstract

The objective of this study was to investigate the effects of simvastatin (SIM) on lipid metabolism disorders and gut microbiota in high-fat diet-induced hyperlipidemic rats. The obtained results revealed that feeding rats with SIM (20 mg/kg/day) significantly decreased serum lipid level and inhibited hepatic lipid accumulation and steatosis. Histological analysis further indicated that SIM reduced lipid deposition in adipocytes and hepatocytes in comparison with that of the HFD group. The underlying mechanisms of SIM administration against HFD-induced hyperlipidemia were also studied by UPLC-Q-TOF/MS-based liver metabonomics coupled with pathway analysis. Metabolic pathway enrichment analysis of liver metabolites with significant difference in abundance indicated that fatty acids metabolism and amino acid metabolism were the main metabolic pathways altered by SIM administration. Meanwhile, operational taxonomic units (OTUs) analysis revealed that oral administration of SIM altered the composition of gut microbiota, including *Ruminococcaceae* (OTU960) and *Lactobacillus* (OTU152), and so on. Furthermore, SIM treatment also regulated the mRNA levels of the genes involved in lipid and cholesterol metabolism. Immunohistochemistry (IHC) analysis of the liver-related proteins (CD36, CYP7A1 and SREBP-1C) showed that oral administration of SIM could regulate the levels of the protein expression related to hepatic lipid metabolism.

## Highlights

1. Simvastatin (SIM) administration could prevent HFD-induced NAFLD associated with hyperlipidemia.
2. SIM administration modulates the intestinal microbiome in HFD-induced hyperlipidemic rats.
3. Relationships between the modulation of intestinal microbiota and hyperlipidemia effect of SIM administration were discussed.
4. The mechanisms of SIM administration against hyperlipidemia were also revealed by liver metabonomics.

## Introduction

Hyperlipidemia is a metabolic disease which is characterized by an abnormal increase in the lipid levels and/or lipoproteins in the blood (Guo et al., 2018). Recently, the prevalence of hyperlipidemia has rapidly increased due to the improved lifestyle and increased consumption of fat or energy-rich diet (Egan et al., 2016). To date, many hypolipidemic drugs have been used to control serum cholesterol levels and to prevent hyperlipidemia. Among these drugs, statins are widely used to retard the atherosclerosis in patients with hyperlipidemia (Macan et al., 2015). In fact, these drugs have also been reported to present anti-inflammatory, anticancer, antioxidant and immunomodulatory effects (Chen et al., 2018; Rizvi et al., 2019). Experiments *in vivo* and *in vitro* have shown that statins protect organism damage from oxidative stress by eliminating superoxide anion and hydroxyl radicals (Murakami et al., 2018). Simvastatin (SIM), a family of statins, is the first choice for the treatment of hypercholesterolemia, dyslipidemia, and coronary heart disease (Rizvi et al., 2019). It is an inhibitor of 3-hydroxy-3-methylglutaryl CoA reductase (HMG-CoA reductase) and responsible for converting HMG-CoA into mevalonate (Harisa et al., 2017). Furthermore, previous studies indicated that SIM could effectively reduce the concentration of atherosclerotic lipoprotein and increase high-density lipoprotein cholesterol (HLD-C) while changing the composition of lipoprotein with a history of mixed hyperlipidemia (Franiak-Pietryga et al., 2009). However, little research has focused on the effects and therapeutic mechanism of SIM *in vivo*.

The gut microbiota is considered as an essential “organ” of the human body, assisting the host's metabolism and protecting immune system (Zhou et al., 2016). It has been well recognized that the composition of the intestinal microbiota had a vital influence on the improvement of lipid metabolic diseases, including nonalcoholic fatty liver disease (NAFLD), hypercholesterolemia, and hyperlipidemia (Tang et al., 2018). Accumulating evidence suggested that the intestinal flora was associated with a variety of metabolic diseases, and the therapeutic responses of clinical drugs (including statins) were through the interaction between drugs and microorganisms (Li and Jia, 2013). Previous studies have shown that rosuvastatin treatment affected the intestinal flora and strengthened the treatment of dyslipidemia (Liu et al., 2018). Kaddurah-Daouk et al. (2011) found that simvastatin metabolism *in vivo* indicated a close correlation with the synthesis of secondary bile acids by microorganisms in the human intestinal tract (Kaddurah-Daouk et al., 2011). This indicated that the bioavailability of simvastatin was closely relevant with the abundance of intestinal flora.

Metabolomics is an important tool for top-down biological systems and is widely used to study drug effects and mechanisms (Li W. et al., 2019). Studies of metabolite concentrations in different tissues or biological fluids have shown that changes in biomarker concentrations may be associated with the corresponding pathways what regulating this change (Miao et al., 2018). The liver plays an indispensable role in multinutrient metabolism, particularly glucose and lipid metabolism, including lipogenesis and cholesterol metabolism (Lin et al., 2017). However, disorders of liver lipid metabolism can contribute to hyperlipidemia, mainly characterized by elevating blood total cholesterol (TC), triglyceride (TG), and low-density lipoprotein cholesterol (LDL-C), and decreasing HDL-C. Liquid chromatography/mass spectrometer (LC/MS) has been applied to analyze metabolomic profiling and biomarker discovery in cells or organisms due to its fast scanning capabilities and accurate mass measurements. In this study, a metabolomic approach was applied to evaluate the antihyperlipidemia effect of SIM and determine the potential hypolipidemic mechanism in rats with SIM administration.

This study aimed to investigate how SIM administration affects lipid metabolism and changes the composition of intestinal microbiota in high-fat diet-induced hyperlipidemic rats by analyzing weight gain, serum and hepatic lipid profile, and morphology of the liver. The intestinal microbiota was analyzed by high-throughput sequencing. The liver metabolites were studied by ultra-performance liquid chromatography-quadrupole time-of-flight mass spectrometry (UPLC-TOF-MS)-based metabonomics coupled with metabolic pathway analysis. This study would offer theoretical evidence for the potential link between bacterial composition and drug-modulation of lipid metabolism.

## Materials and Methods

### Animals and Treatments

Female Sprague-Dawley rats (SD, 8 weeks old, 180–200 g in weight) in the study were purchased from the Shanghai Jake Biotechnology Co., Ltd (Shanghai, China) and placed in a controlled environment (12-h light-dark cycle), temperature (25 ± 1°C) and humidity (55 ± 10%) with free access to food and water. After normal dietary adaptation for one week, the rats were randomly divided into three groups: normal diet (NFD group), high-fat diet (HFD group), and high-fat diet with SIM (SIM group, 20 mg/kg/day). During the feeding period, food intake and the body weight were recorded. SIM group accepted a daily dose of 20 mg/kg/day of SIM through gastrointestinal administration (He et al., 2017), while the NFD group and the HFD group were fed with an equal volume of saline solution for 8 weeks. Animals in all groups were starved for 18 h and anatomized under anesthesia using diethyl ether. The blood samples were drawn from the abdominal vein and put into vacuum tubes. After the organs and adipose tissues were collected, they were immediately frozen at liquid nitrogen and stored at −80°C until use.

### Biochemical Analysis of Serum, Hepatic, and Fecal Samples

Blood samples were collected into tubes after 8 weeks of feeding and then centrifuged for 10 min at 4,000 r/min. The supernatant was collected and frozen at −80°C until use. The liver samples were immediately dissected, weighted and kept in liquid nitrogen, and then stored at −80°C. The lipid profile levels of total cholesterol (TC), triglycerides (TG), low-density lipoprotein cholesterol (LDL-C), high-density lipoprotein cholesterol (HDL-C), total bile acid (TBA), malondialdehyde (MAD), glutathione peroxidase (GSH-PX), total superoxide dismutase (T-SOD), and non-esterified fatty acids (NEFA) in the serum, liver, and feces were quantified using assay kits (Nanjing Jiancheng, Nanjing, China) according to the manufacturer's instructions. The total protein measurement method based on bicinchoninic acid (BCA) also is quantified using assay kits (Nanjing Jiancheng, Nanjing, China) according to the manufacturer's instructions.

### Histopathological Examination

The histomorphological analysis was performed according to the previous published paper with some modifications (Munukka et al., 2017). In brief, after fixation overnight, a portion of the liver tissue was excised and fixed in formalin solution (4%) and embedded in paraffin then cut as 5μm thickness using a manual rotary microtome (LeicaRM2016, Germany). Photomicrographs of hematoxylin–eosin-stained (H&E) sections were obtained with a pathologic slice scanner (Leica DM2700 M, Germany).

### Quantification of Fecal SCFAs

The SCFAs were analyzed according to the previous study with some modifications (Guo et al., 2018). In brief, 50 mg of feces was added to the EP test tube, followed by addition of 500 μl saturated NaCl solution and kept at room temperature for 30 min. The solution was further homogenized for 3 min to dissolve the solid content. Finally, 20 μl of sulfuric acid solution (10%, v/v) was added and vortex concussed for 30 s. The total SCFAs were completely extracted with 800 μl of diethyl ether. This mixture was centrifuged for 15 min (12,000 r/min, 4°C), the supernatant was collected and transferred to another EP test tube, and 0.25 g anhydrous Na_2_SO_4_ was added to remove the trace water. The samples were sequentially centrifuged (1 min, 3,000 r/min), the upper organic layer was filtrated using 0.22 μm filter, and then performed using capillary gas chromatography.

### High Throughput Sequencing of Gut Microbiota

Genomic DNA was extracted from cecal samples using the fecal DNA Isolation Kit and DNA Purification Kit according to the manufacturer's instructions (QIAGEN, Hilden, Germany). The V3–V4 region of the 16S rRNA gene was sequenced using primers 338F (5′-ACT CCT ACG GGA GGC AGC AG-3′) and 806R (5′-GGA CTA CHV GGG TWT CTA AT-3′). Sequencing of the PCR amplification products was performed at the Shanghai Major Bio-Pharm Technology (Shanghai, China) for high throughput sequencing. The raw data from high throughput sequencing that support the findings of this study are openly available at https://www.ncbi.nlm.nih.gov/genbank (Reference number: PRJNA606689).

The relative abundance of gut microbiota at operational taxonomic units (OTUs) level between the NFD and HFD groups or HFD group and SIM group were shown by an extended error bar plot using STAMP (Ver. 2.1.3). The heatmap of correlation between the intestinal microbial phylotypes of significant differences and the biochemical indexes or liver metabolites was implemented by package “pheatmap” through R software (Ver. 3.3.3). The correlation network between lipid metabolism related indexes and the key intestinal microbial phylotypes was created by Cytoscape (Ver. 3.6.0).

### UPLC-QTOF/MS-Based Liver Metabolomics

A total of 25 mg of hepatic sample was weighted in an EP tube, and 1,000 μl extract solution (acetonitrile:methanol:water = 2:2:1) containing internal standard (L-2-Chlorophenylalanine, 2 μg/ml) was added. Then the samples were centrifuged for15 min (10,000 r/min, 4°C). 800 μl of the supernatant was taken to a fresh tube and dried in a vacuum concentrator at 37°C. Then, the dried samples were reconstituted in 200 μl of 50% acetonitrile by sonication on ice for 10 min. The constitution was then centrifuged for15 min (13,000 r/min, 4°C), and 75 μl of the supernatant was transferred to a fresh glass vial for LC/MS analysis. In addition, the quality control (QC) sample was prepared by mixing an equal aliquot of the supernatants from all samples.

The UPLC separation was carried out using a 1290 Infinity series UPLC System (Agilent Technologies), equipped with a UPLC BEH Amide column (2.1 * 100 mm, 1.7 μm, Waters). The mobile phase consisted of 25 mmol/L ammonium acetate and 25 ammonia hydroxide in water (A) and acetonitrile (B). The analysis was carried with elution gradient as follows: 0~0.5 min, 95% B; 0.5~7.0 min, 95%~65% B; 7.0~8.0 min, 65%~40% B; 8.0~9.0 min, 40% B; 9.0~9.1 min, 40%~95% B; 9.1~12.0 min, 95% B. The column temperature was 25°C. The auto-sampler temperature was 4°C, and the injection volume was 2 μl (pos) or 1 μl (neg), respectively.

### Real-Time Quantitative PCR

Liver samples (100 mg) were homogenized in 1 ml of ice-cold RNase plus reagent (TAKARA, Tokyo, Japan), and total RNA was extracted and treated with RNase-free DNase (DNase I; Amersham Pharmacia Biotech Sollentuna, Sweden). Reverse transcription-PCR (RT-PCR) was performed using a TAKARA One-Step RT-PCR kit and rat GAPDH as an internal control gene. RT-PCR was performed as follows: one cycle of cDNA synthesis at 37°C for 15 min and one cycle of initial PCR activation at 95°C for 30 s, followed by 40 cycles of denaturation at 95°C for 5 s, annealing at 55°C for 31 s, and extension at 95°C for 5 s followed by one cycle of a final extension at 72°C for 30 s. Gene expression was normalized to the geometric mean of the reference genes (*β*-ACTIN) using the 2^-ΔΔCt^ method. The primer sequences for real-time qPCR were listed in **Table 1**.

**Table 1:** Primer sequence for quantitative real-time PCR.

Gene	Forward primer(5′–3′)	Reverse primer(5′–3′)
ACAT2	GAACGTGGTGGTCCATGACT	TTCAGCAGACCTCCAACCAC
SREBP-1C	GCTGTTGGCATCCTGCTATC	TAGCTGGAAGTGACGGTGGT
CYP7A1	CACCATTCCTGCAACCTTTT	GTACCGGCAGGTCATTCAGT
CD36	GACAATCAAAAGGGAAGTTG	CCTCTCTGTTTAACCTTGAT
HMGCR	AGTGGTGCGTCTTCCTCG	CGAATCTGCTGGTGCTAT
BESP	CGTGCTTGTGGAAGAAGTTG	GGGAGTAGATGGGTGTGACTG
β-ACTIN	ACGTCGACATCCGCAAAGACCTC	TGATCTCCTTCTGCATCCGGTCA

Fresh rat liver was fixed in 10% neutral-buffered formalin, processed and embedded using an EC360 Tissue Embedder (Meiko, Germany) and cut into 4 um sections for hematoxylin and eosin staining. With regard to immunohistochemistry, hepatic tissue sections were evaluated using the SABC-POD kit (Nanjing Jiancheng, Nanjing, China), and the development localization reaction to primary antibodies to SREBP1, CYP7A1, and CD36 in hepatic tissues, which were then counterstained with hematoxylin. Light microscopy was performed using a Leica B5-223IEP (Leica, Germany).

### Statistical Analysis

Data were presented as mean ± SEM. By means of SPSS 20.0 software (Chicago, IL, USA), the results were analyzed by one-way analysis of variance (ANOVA) among multiple groups followed by Newman Keuls multiple comparison *post hoc* tests. *P* values < 0.05 were considered to be statistically significant.

## Results

### Effects of SIM on Body Weight and Organs Index of HFD-Treated Rats

**Figure 1:**
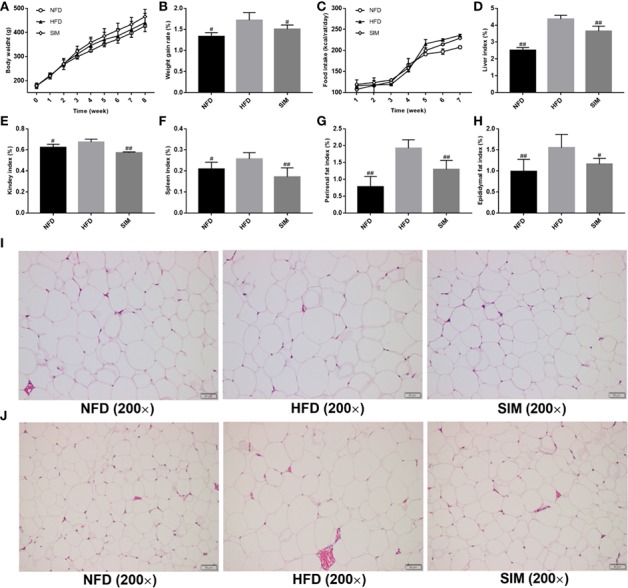
Effects of simvastatin administration on **(A)** body weight, **(B)** body weight gain rate, **(C)** food intakes, **(D)** liver index, **(E)** kidney index, **(F)** spleen index, **(G)** perirenal fat index, **(H)** epididymal fat index, **(I)** the size of perirenal adipocytes, and **(J)** the size of epididymal adipocytes in rats fed a high fat diet. ^#^*P* < 0.05 as compared to the HFD group, ^##^*P* < 0.01 as compared to the HFD group.

As shown in **Figure 1A**, the body weight of the three groups increased with time, especially in the HFD group, while the average body weight gain rate in the SIM group was obviously lower than in the HFD group (**Figure 1B**). The trend of food intake in the NFD group was the highest in all experimental groups (**Figure 1C**). At the end of the treatment, the weight of the liver and adipose tissues increased significantly after HFD feeding compared with the rats in the NFD group. Compared with the HFD group, oral administration SIM helped in preventing abnormal growth of the liver, kidney, and spleen index caused by HFD (*P* < 0.01) (**Figures 1D–F**), indicating the beneficial cure of SIM administration for the liver. It is noteworthy that the perirenal and epididymal fat index of the SIM group was markedly lower than that in the HFD group (P < 0.01) (**Figures 1G, H**). In addition, the microstructure of perirenal fat and epididymal fat tissue was illustrated in all groups (**Figures 1I, J**). The volume and size of perirenal fat and epididymal adipose tissue in the HFD group increased remarkably compared with the NFD group. In contrast, SIM administration effectively prevented abnormal hypertrophy of adipose cells and reduced lipid deposition.

### Effect of SIM on Serum Lipid Level

**Figure 2:**
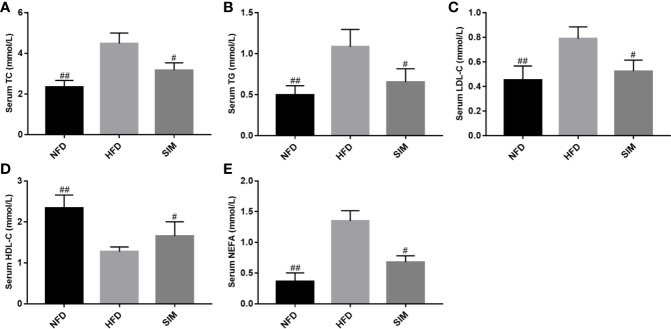
Effects of simvastatin administration on serum **(A)** serum TC, **(B)** serum TG, **(C)** serum LDL-C, **(D)** serum HDL-C and **(E)** serum NEFA levels in rats fed a high fat diet. ^#^*P* < 0.05 as compared to the HFD group, ^##^*P* < 0.01 as compared to the HFD group.

The main characteristic of hyperlipidemia was abnormal levels of serum TC, TG, LDL-C, HDL-C, and NEFA. As shown in **Figure 2**. The HFD group had sharply increased serum TC, TG, LDL-C, and NEFA levels in rats compared with the NFD group (*P* < 0.01). However, after SIM feeding for 8 weeks, there was a significant reduction of serum TG, TC LDL-C, and NEFA levels and improvement of serum HDL-C level compared to those in the HFD group (*P* < 0.05).

### Effects of SIM on HFD-Induced Hepatic Lipid and Pathological Changes

**Figure 3:**
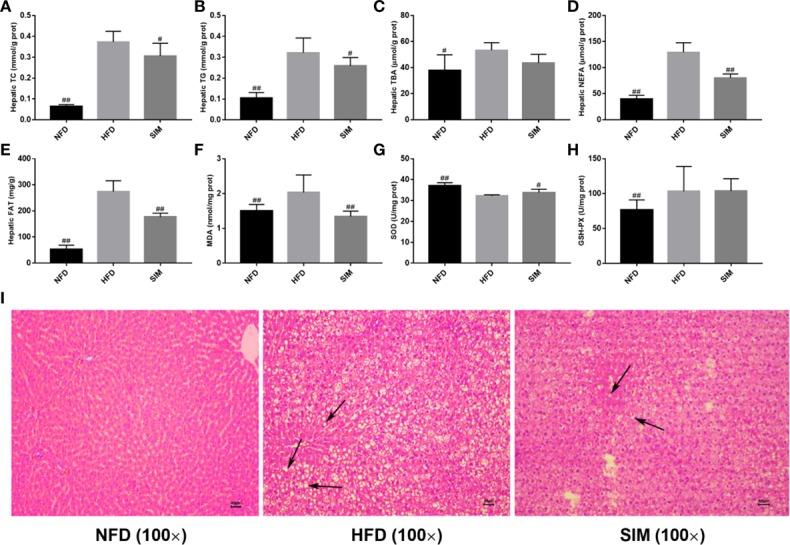
Effects of simvastatin administration on hepatic lipid profile in HFD-fed rats. **(A)** hepatic TC, **(B)** hepatic TG, **(C)** hepatic TBA, **(D)** hepatic NEFA, **(E)** hepatic fat, **(F)** hepatic MDA, **(G)** hepatic SOD, **(H)** hepatic GSH-PX, and **(I)** hepatic morphology (magnification ×100) in rats fed a high fat diet. ^#^*P* < 0.05 as compared to the HFD group, ^##^*P* < 0.01 as compared to the HFD group.

Since the liver is a key tissue in regulation of lipid metabolism, this study further analyzed hepatic TG, TC, TBA, NEFA levels and total fat content (**Figure 3**). As shown in **Figures 3A, B**, compared with the NFD group, the hepatic TG and TC levels were sharply elevated after the high-fat diet for 8 weeks (*P* < 0.01). However, oral administration of SIM effectively prevented accumulation of TC or TG in liver (*P* < 0.05). Moreover, administration of SIM dramatically decreased hepatic NEFA and fat, compared to the HFD group (*P* < 0.01). In addition, the effect of SIM on hepatic oxidative stress levels in high-fat rats was shown in **Figures 3F–H**. The result showed that SIM feeding sharply reduced MDA levels and increased SOD activities in the liver. Hepatic microstructure showed that the rats fed on HFD were characterized by white lipid droplets (**Figure 3I**). The high-fat diet significantly increased the accumulation of fat compared to the NFD group; however, white lipid droplets in the SIM group were less than those in the HFD group. Furthermore, the lipid droplets and inflammatory cells of the SIM group were reduced as compared with the HFD group, indicating that SIM can reduce the accumulation of lipids and have a protective effect on the liver.

### Effects of SIM on Lipid Excretion and SCFA Production in Feces

**Figure 4:**
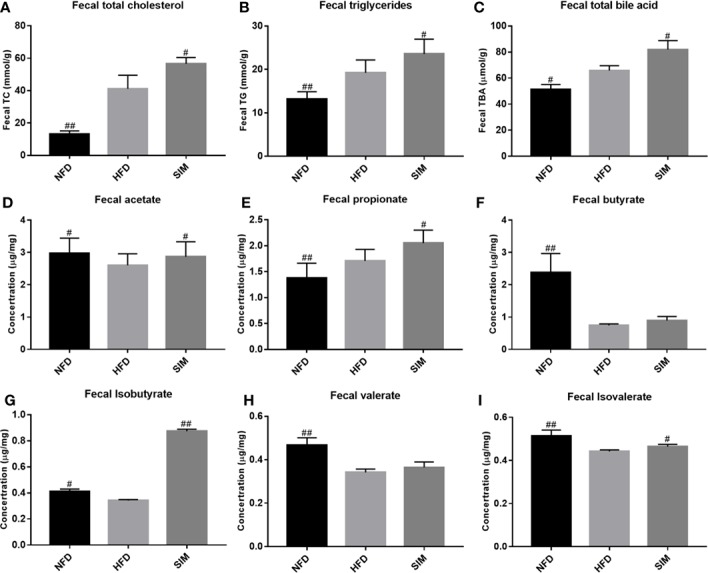
Effect of simvastatin administration on the fecal lipid levels and short-chain fatty acids (SCFAs) levels. **(A)** fecal TC, **(B)** fecal TG, **(C)** fecal TBA, **(D)** fecal acetate, **(E)** fecal propionate, **(F)** fecal butyrate, **(G)** fecal isobutyrate, **(H)** fecal valerate, and **(I)** fecal isovalerate levels. ^#^*P* < 0.05 as compared to the HFD group, ^##^*P* < 0.01 as compared to the HFD group.

The effects of SIM administration on fecal lipid excretion were shown in **Figure 4**. After the consumption of high-fat diets, the levels of fecal TC and TG in the HFD group sharply increase compared with those in the NFD group (*P* < 0.01). However, SIM feeding enhanced lipid excretion (fecal TC, TG, and TBA), which indicated that oral administration of SIM could effectively promote the excretion of fecal lipid (fecal TC, TG, and TBA) through the intestinal tract of rats. Short chain fatty acid (SCFA) concentration in feces was shown in **Figures 4D–I**. Compared with the NFD group, high-fat diet produced higher SCFA levels in rats, while SIM administration significantly increased the levels of fecal acetate, propionate, isobutyrate, and isovalerate in rats, especially for fecal isobutyrate (*P* < 0.01).

### Effects of SIM on the Composition of Intestinal Microflora

**Figure 5:**
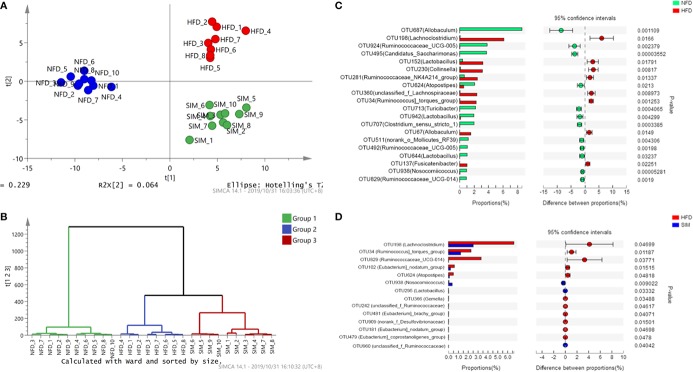
The overall structural changes of the gut microbiota were analyzed among different groups. **(A)** Principal component analysis and **(B)** hierarchical clustering tree analysis. Extended error bar plot comparing the differences in the mean proportions of the significantly altered intestinal microbial phylotypes. **(C)** NFD group *versus* HFD group, **(D)** SIM group *versus* HFD group.

To investigate structural changes of gut microbiota on SIM administration to rats, the alpha diversity of gut microbiota analyzed using Simpson and Shannon index reveals a significant difference among the NFD, HFD, and SIM groups (**Table 2**). The Shannon index and Simpson index reflected the heterogeneity in the microbiome. The results revealed that a significant difference in alpha diversity was spotted by Shannon index (*P* < 0.05) and Simpson index (*P* < 0.05) of the HFD and SIM groups' gut microbiome. Furthermore, the abundance of gut microbiome in the SIM group is similar to the NFD group. XM_FUniFrac distance-based principal component analysis (PCA) was used to examine the structural changes of intestinal microflora (**Figure 5A**) and hierarchical clustering tree analysis (**Figure 5B**). PCA score plot indicated that the organismal structure of the gut microbiota in the HFD group rats clearly separated from the NFD group (**Figure 5A**). However, administration of SIM altered the high-fat diet-induced variations, which was similar to that of the NFD group. The hierarchical clustering plot also showed the same tendency (**Figure 5B**). In general, oral administration SIM has a significant influence on improving the composition of intestinal microflora in rats induced by HFD.

**Table 2:** Quantity of operational taxonomic unit (OTU) and Simpson and Shannon index of bacterial diversity in normal diet (NFD) group versus high-fat diet (HFD) group and HFD group versus simvastatin (SIM) group (n=8, x ± sd).

Treatment	OTU level	Alpha-diversity	
		Simpson	Shannon
NFD	40,833 ± 7,698^#^	0.076 ± 0.015^#^	3.87 ± 0.20^#^
HFD	37,782 ± 4,216	0.052 ± 0.015	3.56 ± 0.17
SIM	40,712 ± 6,458^#^	0.14 ± 0.086^#^	3.01 ± 0.47^#^

^#^P < 0.05 as compared to the HFD group.

**Table 3** shows the differences of OTU quantity among the NFD, HFD, and SIM groups. The relative abundance of identified OTUs was analyzed among the three groups (**Figures 5C, D**). Among the predominant OTUs, there was obviously a phenomenon that the level of *Ruminococcus]_torques_group* (OTU34), *Lachnoclostridium* (OTU198)*, Lactobacillus* (OTU152), and *Collinsella* (OTU230) increased in the HFD groups. *Ruminococcaceae_UCG-005* (OTU924), *Candidatus_Saccharimonas* (OTU495), *Atopostipes* (OTU624), *Lactobacillus* (OTU942), *Clostridium_sensu_stricto_1* (OTU707), and *Ruminococcaceae_UCG-005* (OTU492) tended to be increased in the NFD group. Compared with those in the HFD group, *Nosocomiicoccus* (OTU938), *Lactobacillus* (OTU295), and *unclassified_f_Ruminococcaceae* (OTU960) tended to be higher levels after administration of SIM. However, the numbers of *[Eubacterium]_nodatum_*group (OTU102) and *Atopostipes* (OTU624) were decreased significantly.

**Table 3:** Potential biomarkers in liver associated with SIM administration based on ultra-performance liquid chromatography-quadrupole time-of-flight mass spectrometry (UPLC-QTOFMS).

ion mode	RT-Min	Metabolite	Mass	VIP	P-value	Related pathway	Changes^a^
Negative	97.57	Docosahexaenoic acid	330.08	1.48	0.05	Biosynthesis of unsaturated fatty acids	↑
	209.14	Hypotaurine	108.02	1.80	0.01	Taurine and hypotaurine metabolism	↑
	283.25	Taurine	124.01	1.34	0.01	Primary bile acid biosynthesis	↑
	41.523	Arachidonic Acid	607.47	1.90	0.00	Biosynthesis of unsaturated fatty acids	↑
	344.99	Glyceric acid	166.04	1.84	0.01	Glycerolipid metabolism	↑
	42.817	Linoleic acid	279.23	1.60	0.02	Biosynthesis of unsaturated fatty acids	↑
Positive	47.16	20-Hydroxyarachidonic acid	321.24	1.67	0.01	–	↑
	127.58	Taurodeoxycholic acid (TDCA)	464.28	1.81	0.01	–	↑
	200.89	Chenodeoxyglycocholic acid (CDCA)	414.30	1.08	0.04	–	↓
	201.02	Glycodeoxycholic acid (GDCA)	450.32	1.12	0.04	–	↓
	249.15	3-(2-Hydroxyphenyl) 4-propionic acid	138.05	1.84	0.00	Propanoate metabolism	↑

a The differential metabolites were determined with the criteria of Variable Importance for the Projection (VIP) > 1 in partial least squares-discriminate analysis (PLS-DA) model of multivariate statistical analysis and P < 0.05 in univariate statistical analysis. ↑, ↓ All changes are significant (P < 0.05).

### Correlation Between Gut Microbiota and Lipid Metabolism Associated Parameters

**Figure 6:**
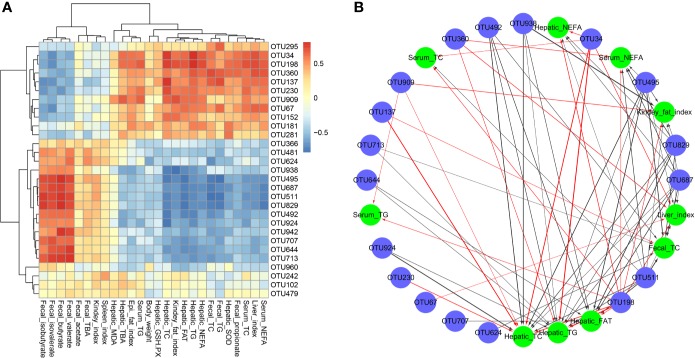
Spearman's correlations between the cecal microbiota and lipid metabolic parameters. **(A)** Heatmap of correlation coefficients and **(B)** visualization of the correlation network between the intestinal microbial phylotypes of significant differences and lipid metabolic parameters.

The correlation between intestinal microbiota and hyperlipidemia related parameters was investigated based on the heatmap (**Data Sheet 1**) and network analysis. Interestingly, a clear correlation with the hyperlipidemia related parameters was found for the regulated intestinal microbiota at the genus level (**Figures 6A, B**). The hepatic TC, TG levels and total fat content and the fecal TC level showed negative correlations with *Candidatus_Saccharimonas* (OTU495), *Ruminococcaceae_UCG-005* (OTU492), and *Norank_o_Mollicutes_RF39* (OTU511) but correlated positively with *Ruminococcus]_torques_group* (OTU34) and *Lachnoclostridium* (OTU198). In addition, *Ruminococcaceae* (OTU960) positively correlated with the intestine SCFAs (including fecal butyrate, valerate, and isobutyrate). Heatmap analysis showed that *Lactobacillus* (OTU152) was positively correlated with fecal indicators (fecal TG and TC) and hepatic antioxidant activity (hepatic SOD and GSH-PX). In short, it sought to indicate that SIM was beneficial to inhibit HFD-induced hyperlipidemia by improving the dysbiosis of the intestinal microbiota.

### Metabolomics Analysis of the Liver After SIM Treatment

**Figure 7:**
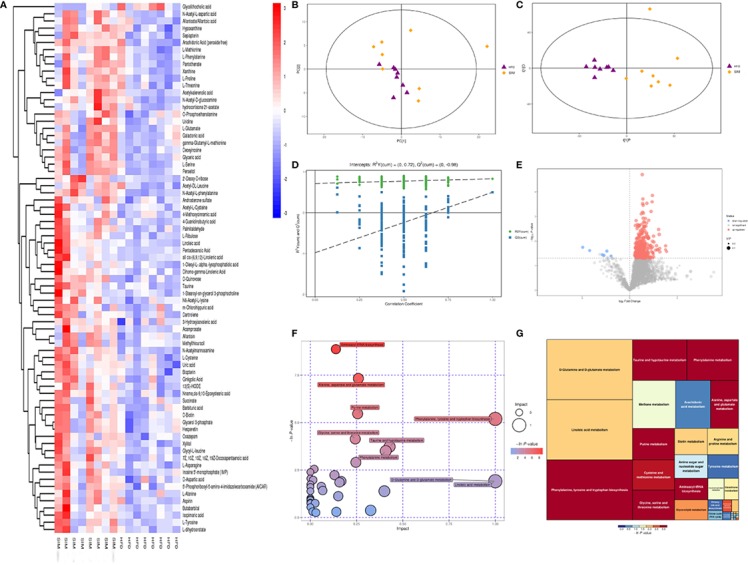
Liver metabolomic profiling by UPLC-QTOF MS in negative-ion modes. **(A)** Heatmap of HFD and SIM groups to visualize the abundance of biomarkers in each group, **(B)** PCA score plot (HFD and SIM groups), **(C)** PLS-DA score plot of HFD and SIM groups, **(D)** loading spots derived from the OPLS-DA models represent the potential markers which are far away from the ion center, **(E)** permutation test from PLS-DA models, **(F)** metabolic pathway analysis based on potential biomarkers of the SIM group compared with those in the HFD group, and **(G)** the metabolic pathway impact prediction between the HFD and SIM groups in the liver based on KEGG online database. The -ln(p) values from the pathway enrichment analysis are indicated on the horizontal axis, and the impact values are indicated on the vertical axis.

**Figure 8:**
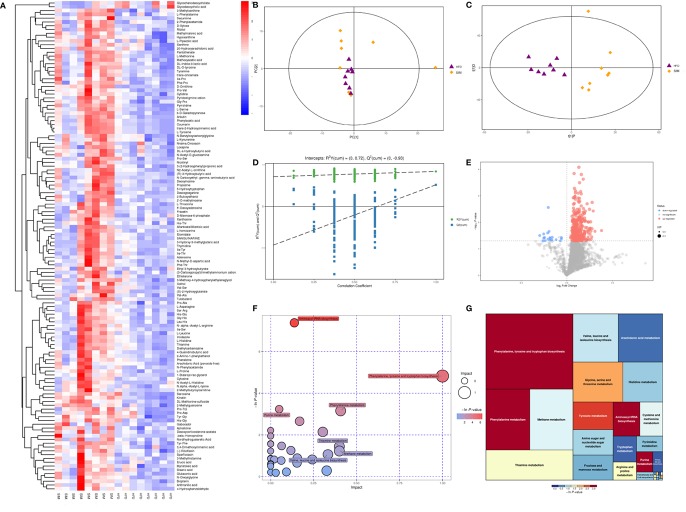
Liver metabolomic profiling by UPLC-QTOF MS in positive-ion modes. **(A)** Heatmap of HFD and SIM groups to visualize the abundance of biomarkers in each group, **(B)** PCA score plot (HFD and SIM groups), **(C)** PLS-DA score plot of HFD and SIM groups, **(D)** Loading spots derived from the OPLS-DA models represent the potential markers which are far away from the ion center, **(E)** permutation test from PLS-DA models, **(F)** metabolic pathway analysis based on potential biomarkers of the SIM group compared with those in the HFD group. **(G)** The metabolic pathway impact prediction between the HFD and SIM groups in the liver based on KEGG online database. The -ln(p) values from the pathway enrichment analysis are indicated on the horizontal axis, and the impact values are indicated on the vertical axis.

Using principal component analysis (PCA) and partial least squares-discriminate analysis (PLS-DA), distinct changes in metabolite patterns in the liver were observed (**Figures 7**, **8**). The PLS-DA score plot demonstrated that the metabolic profiles of the HFD group rats were segregated well from those of the SIM group rats, indicating that SIM treatment may cause significant biochemical changes in the liver. Moreover, the OPLS-DA parameters with a high explanation rate (R^2^X = 0.72) and a high prediction rate (Q^2^ = 0.98) for negative data (**Figure 7**) and R^2^X = 0.72, Q^2^ = 0.93 for positive data (**Figure 8**) illustrated an excellent predictive power. In this study, the metabolites with VIP value > 1.0 and *P* value < 0.05 were potential biomarkers, which were responsible for the differences between the HFD and SIM groups. Based on the UPLC-QTOF/MS data in the negative-ion mode, we identified a total of 74 potential biomarkers (**Data Sheet 2**) in the liver between the HFD and SIM groups (**Figure 7A**), of which 73 metabolites were significantly elevated and one metabolite was significantly decreased in the SIM group compared with those in the HFD group. A total of 129 potential biomarkers (**Data Sheet 3**) in the liver were successfully identified in positive-ion mode (**Figure 8A**) compared with the HFD group, 127 metabolites were significantly up-regulated and two metabolites were significantly down-regulated in the SIM group.

To acquire some deeper understanding of metabolic changes in response to the intervention of SIM in hyperlipidemic rats, metabolic pathway enrichment analysis of the differential hepatic metabolites was performed by MetaboAnalyst 4.0 (https://www.metaboanalyst.ca/) based on KEGG (http://www.genome.jp/kegg/) to determine the important metabolic pathways that were affected. In the negative-ion mode, the metabolic pathways altered by SIM treatment compared with the HFD-fed hyperlipidemic rats mainly included D-glutamine and D-glutamate metabolism, linoleic acid metabolism, phenylalanine, tyrosine and tryptophan biosynthesis, taurine and hypotaurine metabolism, phenylalanine metabolism, methane metabolism, arachidonic acid metabolism, primary bile acid biosynthesis, *etc*. (**Figures 7F, G**). In the positive-ion mode, metabolic pathway enrichment result indicated that phenylalanine, tyrosine and tryptophan biosynthesis, phenylalanine metabolism, methane metabolism, thiamine metabolism, valine, leucine and isoleucine biosynthesis, arachidonic acid metabolism, glycine, serine and threonine metabolism, *etc*. were the main metabolic pathways altered by SIM intervention when compared against hyperlipidemic rats of the HFD group (**Figures 8F, G**).

### Correlation Analysis of Gut Microbiota and Liver Metabolites

**Figure 9:**
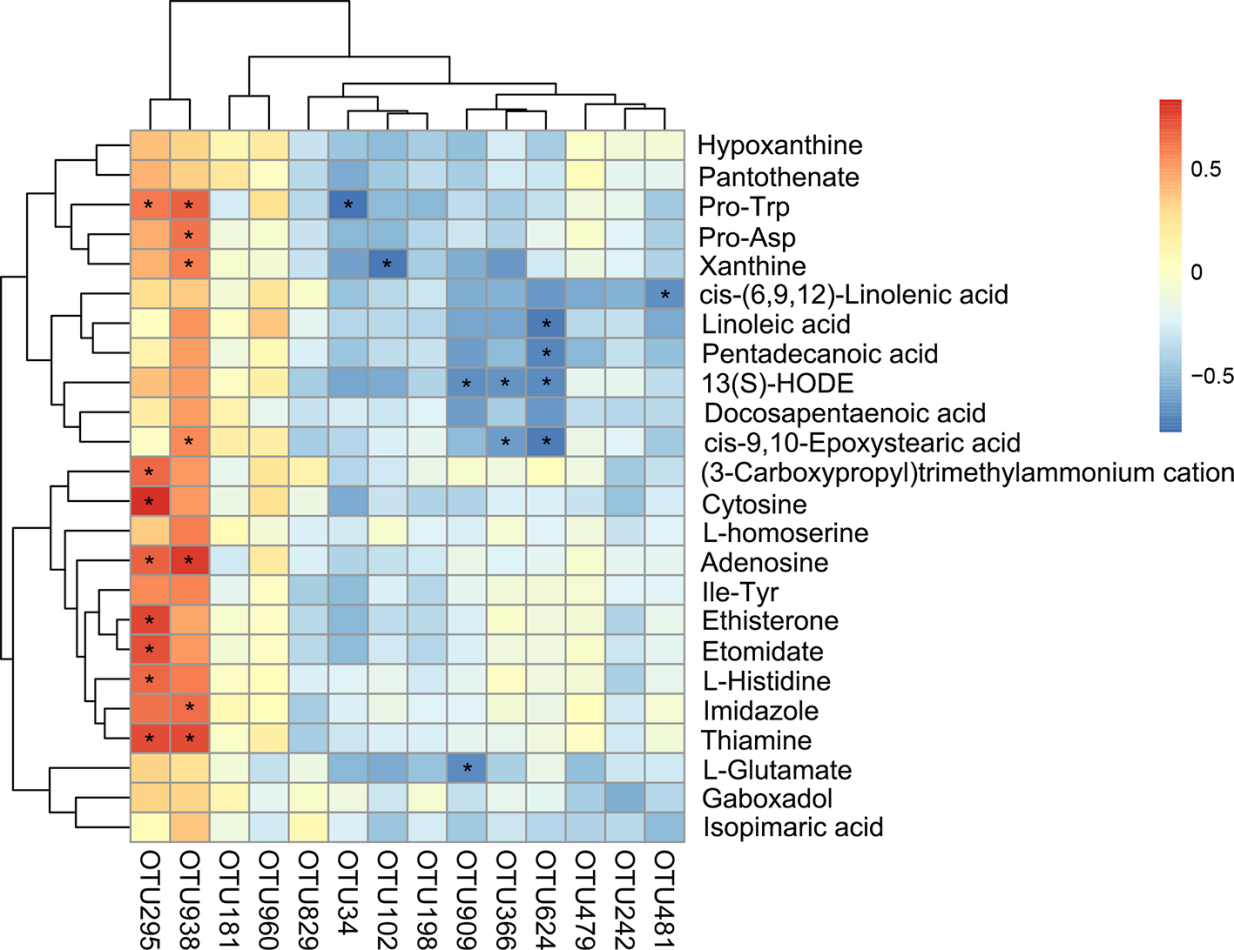
Statistical Spearman's correlations between the intestinal microbial phylotypes and liver metabolites of significant differences.

The correlation between the intestinal microbiota and liver metabolites was investigated based on heatmap (**Figure 9**) (**Data Sheet 4**). *Lactobacillus* (OTU295) and *Nosocomiicoccus* (OTU938) showed positive correlations with Pro-Trp, adenosine, and thiamine. Particularly, *Lactobacillus* (OTU295) was also positively correlated with L-histidine, ethisterone, etomidate, cytosine, and (3-carboxypropyl) trimethylammonium cation. Meanwhile, *Nosocomiicoccus* (OTU938) was also positively correlated with xanthine and cis-9,10-epoxystearic acid. However, [Eubacterium]_nodatum_group (OTU102) negatively correlated with xanthine. In addition, *Atopostipes* (OTU624) correlated negatively with linoleic acid, pentadecanoic acid, 13(S)-HODE, and cis-9,10-epoxystearic acid.

### Effects of SIM on mRNA Levels Responsible for Hepatic Lipid Metabolism and Immunohistochemistry Analysis of Protein Expression

**Figure 10:**
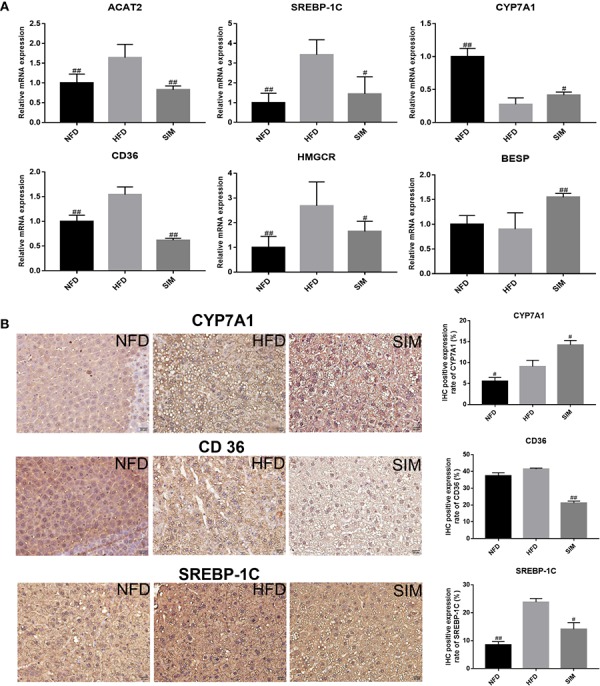
Effects of simvastatin administration on the expression of hepatic related genes in HFD-fed rats. The bar graphs showed mRNA levels of **(A)** ACAT2, SREBP-1C, CYP7A1, CD36 HMGCR, and BESP, which were determined by RT-qPCR. **(B)** Immunohistochemical staining for CYP7A1, CD36, and SREBP-1C proteins in the in liver tissue; brown color (positive) indicates cells expressing CYP7A1, CD36, and SREBP-1C proteins (scale bars = 20 μm). Paraffin sections slightly counterstained with hematoxylin. Quantification of CYP7A1, CD36, and SREBP-1C expression by IHC was also shown on the right. ^#^*P* < 0.05 as compared to the HFD group, ^##^*P* < 0.01 as compared to the HFD group.

To understand the mechanisms of SIM antihyperlipidemia, the effect of mRNA expression (ACAT2, SREBP-1C, CYP7A1, CD36, HMGCR and BESP) in rats' liver and genes related to hepatic lipid metabolism were represented in **Figure 10A**. The expression of target genes in the liver was examined by RT-PCR. The expression of BESP and CYP7A1 in the SIM group was up-regulated, and ACAT2, SREBP-1C, CD36, and HMGCR levels were down-regulated relative to those of the HFD group. The results of immunohistochemistry (IHC) analysis of the protein expressions of CD36, CYP7A1, and SREBP-1C in the liver samples are presented in **Figure 10B**, indicating that high-fat diet was higher than normal diet, but SIM administration up-regulated the mRNA and protein expression of CYP7A1 and suppressed CD36 and SREBP-1C expression in the liver. These results were consistent with the hepatic mRNA levels investigated by RT-qPCR.

## Discussion

Hyperlipidemia has been recognized as a prevalent metabolic disease, mainly due to long-term consumption of high-fat diet (Lv et al., 2019). SIM as a hypolipidemic drug has been widely employed for the treatment of lipid metabolism disorders, including hyperlipidemia, hypercholesterolemia (Miller et al., 2001; Krysiak et al., 2014). While most efforts to understand SIM have focused on genetic polymorphisms (Catry et al., 2015) and pharmacokinetics (Lu et al., 2018), recent study revealed that certain pharmacotherapies can improve metabolic diseases through modulation of the gut microbiota (Yoo et al., 2014). However, the composition of the gut microbiota in response to hypolipidemic effect of SIM has not yet been fully investigated. In this study, high-throughput sequencing was used to elucidate the gut microbiota compositions in high-fat rats that respond positively to SIM treatment. We observed that oral administration of SIM profoundly prevents HFD-induced hyperlipidemia and ameliorates gut microbiota dysbiosis in hyperlipidemic rats. Our current results showed that simvastatin administration could regulate the disorders of lipid metabolism in a hyperlipidemic rat model fed with HFD. Moreover, we found that SIM administration significantly inhibited the excessive weight gain caused by a high-fat diet. Interestingly, SIM resulted in the beneficial effects in hyperlipidemic rats by regulating abnormal serum and liver lipid levels. A similar study indicated that the levels of TC and LDL-C could be regulated by simvastatin in patients with coronary heart disease (Zhang et al., 2013). Oikonomidis et al. found that simvastatin treatment in the cholesterol diet rabbits was beneficial for hypolipidemia and antiatherosclerosis by decreasing serum TC, LDL-C, and TG levels (Oikonomidis et al., 2016). Simvastatin treatment also regulated cholesterol synthesis and ameliorated the lipid droplets accumulation and steatosis in the liver. These results were consistent with those found in previous studies (Musso et al., 2016; Li et al., 2017). High-fat diet induces the formation of free radicals leading to lipid peroxidation and oxidative stress. A previous study showed that antioxidant activities may play a role in the modulation of lipid peroxide level and *in vivo* antioxidant status (Chan et al., 2013). In this study, high-fat diet caused a significant change in hepatic MDA concentrations, SOD activities in rats, while SIM administration obviously improved the antioxidant activity. Moreover, oral administration of SIM enhanced the fecal levels of TC and TG and BAs. The hepatic TC is converted to BAs *via* the “classic” and “alternative” pathways, and then the BA enterohepatic circulation participates in several hepatic and gastrointestinal physiological functions and increased the fecal bile acid excretion (Trauner et al., 2017).

Metabonomics is a new science to provide quantitative measures of metabolic changes and further enable identification of biomarkers in organisms throughout the experiment. These biomarkers could reflect disease diagnosis, metabolic characteristics and reveal metabolic mechanisms (Li et al., 2016). The ability of the liver for lipid metabolism is vital to account for the clearance of dietary lipid. So we applied integrated metabolomics in the liver to identify a number of metabolites in hyperlipidemic rats administrated with SIM. From the results of liver metabolomics, SIM administration had outstanding therapeutic effects on improving lipid metabolism, including fatty acid metabolism and primary bile acid biosynthesis.

Docosahexaenoic acid (DHA), arachidonic acid (AA), and linoleic acid (LA) are polyunsaturated fatty acids (PUFAs), which had been confirmed to have a beneficial effect on cardiovascular disease (Zhao et al., 2019). AA was reported as “beneficial in improving cardiovascular system function”. In the present study, levels of DHA and AA in the administration of SIM were up-regulated compared with the HFD rats. Furthermore, related evidence verified that oral statins decreased concentration of cholesterol and increased arachidonic acid synthesis (Altmaier et al., 2014). Previous evidence has confirmed that patients with cardiovascular disease have lower AA concentration, and increased AA level may reduce cardiovascular risks (Das, 2008). LA is an essential fatty acid (EFA), and positively regulates lipid metabolism by lowering serum TC and LDL-C levels to achieve against cardiovascular risk (Xu et al., 2014). LA also is a precursor of prostaglandins (PGs) *via* biosynthesis of unsaturated fatty acids. PGs have many beneficial effects against hyperlipidemia (Russo, 2009). In our study, SIM administration caused a significant increase in linoleic acid level (P < 0.05) compared with the HFD group.

Bile acids are amphipathic molecules that are using cholesterol as a raw material and end products of cholesterol metabolism in the liver (Jang et al., 2018). Taurine is one of the important sulfonic acid amino acids in all mammalian tissues and has very important physiological functions such as bile acid conjugation, antioxidant and anti-inflammatory activity and ATP production (Zhou et al., 2015). Previous research showed that taurine was able to improve insulin sensitivity and hyperlipidemia because taurine is required for bile acid conjugation, which is lost in the excreta, and then the level of taurine will be decreased. However, SIM administration significantly increases the level of taurine in the liver, indicating that SIM can counteract the negative effect of hyperlipidemia on taurine formation. These primary BAs preferentially activate FXR rather than TGR5, which caused increased glycogenesis and decreased gluconeogenesis (Wang et al., 2018). GCDCA is considered as the main toxic component of BAs that plays a prominent part in hepatocyte apoptosis resulting in cholestatic liver injury (Schoemaker et al., 2003). Our data show that SIM could sharply down-regulate the hepatic GCDCA level, indicating a beneficial effect against the hepatotoxicity of GCDCA. TDCA is a main bioactive substance in animal BAs that is found to have the potential of anti-inflammatory activity (Ren et al., 2019). Prior investigation reported that TDCA might significantly inhibit acute myocardial infarction and chronic inflammation by activating protease cascades to induce apoptosis (Mao et al., 2018). Our results indicated higher TDCA level with SIM administration, which was concordant with increased TDCA levels in improving glucose-lipid metabolism and the pool of BAs (Wu et al., 2013).

Accumulating evidence showed that the changes of the gut microbiota played an important role in the regulation of liver function and the development of metabolic disorders, such as diabetes and hyperlipidemia (Li L. et al., 2019). Clinical studies have found a strong link between the gut microbial composition and host lipid metabolism. At the OTU level, the abundance of *Ruminococcaceae_UCG-005* (OTU492) and Ruminococcaceae_UCG-014 (OTU829) was significantly decreased due to HFD; however, after SIM administration the abundance of *Ruminococcaceae* (OTU960) was obviously increased. Ruminococcaceae of Firmicutes phylum is probiotics known to exert health-promoting effects on the host intestine. In recent years, probiotic bacteria have been regarded as potential biotherapeutics for hyperlipidemia (Kumar et al., 2012). Furthermore, Spearman's correlation analysis showed that the richness of *Ruminococcaceae* (OTU960) negatively correlated with host liver metabolic indicators (hepatic TC, TG and FAT and fecal TC). This is consistent with previous research that the *Ruminococcaceae family* has been confirmed to decrease the levels of triacylglycerols, phospholipids, and cholesteryl esters (Munukka et al., 2017). Previous study revealed that *Ruminococcus* was the short-chain fatty acid (SCFA) producer (Zhou et al., 2019). In this study, we found that *Ruminococcaceae* (OTU960) positively correlated with the intestinal SCFAs (including fecal butyrate, valerate, and isobutyrate). Moreover, *Lactobacillus* spp. have been confirmed to reduce cholesterol availability for gut absorption by assimilation (Kaddurah-Daouk et al., 2011), by binding it to the bacterial cellular surface, or by incorporation into the bacterial membranes (Catry et al., 2015). This conclusion is consistent with our research results, indicating that SIM administration significantly increased the abundance of *Lactobacillus* (OTU152), which was significantly positively correlated with obesity-related indicators (fecal TG and TC). Previous study showed there was a negative correlation in the good/poor response to simvastatin therapy for patients with secondary bile acids produced by intestinal bacteria (Kaddurah-Daouk et al., 2011). These enteric bacteria-produced bile acids included lithocholic acid (LCA) and the conjugated derivatives glycolithocholic acid (GLCA) and taurolithocholic acid (TLCA). LCA is derived from CDCA by intestinal bacteria of *Clostridium*, which is identified as a marker for good response to simvastatin treatment. He et al. (2017) pointed out that the lipid-lowering effect of SIM may be achieved through the regulation of bile acid metabolism of gut microbiota.

To further elucidate the efficacy of SIM administration against lipid metabolism disorders, hepatic mRNA expression levels of BESP, CYP7A1, ACAT2, SREBP-1C, CD36, and HMGCR are investigated. Previous study showed that SREBP-1C was a key factor involved in the transcription of cholesterol and modulation of aliphatic acid synthesis (Peng et al., 2018). Oral administration of SIM can significantly reduce the transcription level of SREBP-1C compared with the HFD group. This result indicates that SIM may regulate hepatic lipid metabolism disorder by inhibiting SREBP-1C. HMGCR is the rate-limiting enzyme in the biosynthesis of cholesterol, and its mRNA expression is decreased in response to SIM suppressing biosynthesis of fat and cholesterol (Li X. et al., 2019). Similarly, emerging evidence showed that inhibition of hepatic HMGCR by statins led to reduce expression of LDL receptors and to further increase the uptake of circulating LDL particles (Würtz et al., 2016). In contrast, CYP7A1 is the first and key enzyme in the process of transformation from cholesterol to bile acids and determining the size of BA pool in the liver (Xie et al., 2019). Up-regulation of CYP7A1 expression can prevent the accumulation of cholesterol in the liver by accelerating the conversion of cholesterol into BAs (Sun et al., 2019). This result suggested that SIM may enhance bile acid synthesis by stimulating CYP7A1. The cholesterol absorbed by the liver was converted into bile acids by a series of enzymes, which excreted the duodenum through the tubules and the basolateral side (Song et al., 2016). Expression of CYP7A1 gene can be sharply inhibited by high-fat diet feeding and greatly stimulated by simvastatin treatment (He et al., 2017). BESP is responsible for excretion of bile acids into the bile canaliculus. In addition, CD36 is key transporter of free fatty acid and has an important influence on the development of NAFLD (Zhao et al., 2018). Previous evidence demonstrated that regulating CD36 expression in the liver could be related to insulin resistance and also prevented lipid accumulation of rats fed a high-fat diet (Khan and Kowluru, 2018; Zhang et al., 2018). Our results suggested that treatment with SIM significantly reduced the hepatic CD36 levels and significantly increased the hepatic BESP levels.

## Conclusion

This study reported that the therapeutic outcomes of simvastatin against high-fat diet induced hyperlipidemia in a rat model. The beneficial effects may be associated with hepatic lipid metabolism by influencing relational mRNA expression and protein expression levels and also the modulation of the intestinal microflora. Hepatic untargeted metabonomics revealed that fatty acid metabolism and bile acid biosynthesis were mainly regulated by SIM administration. Moreover, changes in the intestinal microflora could affect the hepatic lipid metabolism and SCFAs-dependent pathways. Of course, the in-depth underlying mechanism of SIM against hyperlipidemia needs to be further explored through hepatic proteomics and metagenomics of intestinal microflora and even through fecal microbiota transplantation to verify the role of specific intestinal microbial phylotypes under SIM administration.

## Data Availability Statement

The sequencing data generated in this study has been deposited into the Sequence Read Archive database (accession: SRP249560).

## Ethics Statement

All animal experiments were carried out in strict accordance with the ARRIVE guidelines, the National Institutes of Health guide for the care and use of Laboratory animals (NIH Publications No. 8023, revised 1978). All experimental procedures were approved by the animal ethics committee of College of Food Science, Fujian Agriculture and Forestry University (No. FAFU-2019-053).

## Author Contributions

QZ, XF, etc. are responsible for the operation of the experiment, data processing, and writing. RY, YH, TZ, RS, etc. are responsible for interpretation of data and revising it critically. XL, WC, LC, PL, etc. are responsible for the design of the work, program development, writing guidance of the experimental program, and revising it critically for important intellectual content.

## Funding

This work was supported by the Research and Application of New Technologies for the Utilization of Marine Living Resources (CXZX2017017), National Natural Science Foundation of China (Grant No. 31801465), and Outstanding Young Scientiﬁc Research Talent Program of Fujian Agriculture and Forestry University (xjq201808).

## Conflict of Interest

The authors declare that the research was conducted in the absence of any commercial or financial relationships that could be construed as a potential conflict of interest.
